# Predictions of Experimentally Observed Stochastic Ground Vibrations Induced by Blasting

**DOI:** 10.1371/journal.pone.0082056

**Published:** 2013-12-17

**Authors:** Srđan Kostić, Matjaž Perc, Nebojša Vasović, Slobodan Trajković

**Affiliations:** 1 Department of Geology, University of Belgrade Faculty of Mining and Geology, Belgrade, Serbia; 2 Department of Physics, Faculty of Natural Science and Mathematics, University of Maribor, Maribor, Slovenia; 3 Department of Applied Mathematics, University of Belgrade Faculty of Mining and Geology, Belgrade, Serbia; 4 Department of Underground Mining, University of Belgrade Faculty of Mining and Geology, Belgrade, Serbia; University of Warwick, United Kingdom

## Abstract

In the present paper, we investigate the blast induced ground motion recorded at the limestone quarry “Suva Vrela” near Kosjerić, which is located in the western part of Serbia. We examine the recorded signals by means of surrogate data methods and a determinism test, in order to determine whether the recorded ground velocity is stochastic or deterministic in nature. Longitudinal, transversal and the vertical ground motion component are analyzed at three monitoring points that are located at different distances from the blasting source. The analysis reveals that the recordings belong to a class of stationary linear stochastic processes with Gaussian inputs, which could be distorted by a monotonic, instantaneous, time-independent nonlinear function. Low determinism factors obtained with the determinism test further confirm the stochastic nature of the recordings. Guided by the outcome of time series analysis, we propose an improved prediction model for the peak particle velocity based on a neural network. We show that, while conventional predictors fail to provide acceptable prediction accuracy, the neural network model with four main blast parameters as input, namely total charge, maximum charge per delay, distance from the blasting source to the measuring point, and hole depth, delivers significantly more accurate predictions that may be applicable on site. We also perform a sensitivity analysis, which reveals that the distance from the blasting source has the strongest influence on the final value of the peak particle velocity. This is in full agreement with previous observations and theory, thus additionally validating our methodology and main conclusions.

## Introduction

Blasting is commonly performed for hard rock excavation activities, especially in mining and quarrying, but also in tunnel, subway, highways and dam construction [1]. When an explosive charge detonates in a blast hole, the seismic waves transmitted to the surrounding rock set up a ground motion [2], which can be strong enough to cause damage to buildings and other man-made structures [3]. As ground motion directly affects structural responses, it is very important to study its characteristics in order to assess the effects of ground vibrations on structures more reliably [4]–[5].

Common practice uses peak particle velocity (PPV) to predict structural responses [6]. For example, severe damage will occur if PPV exceeds 6 or 23 cm/s for structures located on soil or rock site, respectively. Some other criteria that relate the structural response and damage to both PPV and dominant ground motion frequency, give allowable PPV as frequency dependent [7]–[8]. In practice, the value of PPV is usually estimated using various empirical ground motion attenuation relations [4], [9]–[10]. These equations are of great interests for field engineers, since they enable them to predict the maximum ground vibration, depending on the number of parameters like maximum charge per delay or distance from the blasting source [2], [6], [11]–[14]. However, in spite of the existing and widely used deterministic engineering models of blast-induced ground motion, there is still lack of direct experimental evidence confirming its stochastic or deterministic nature.

In this paper, our aim is to examine the nature of the behavior of the blasting induced ground motion by applying methods of nonlinear time series analysis [15]. In particular, we first wish to determine whether it is deterministic or stochastic, as this imposes critical guidelines for further analysis. In [16] it was shown that earthquake ground motion recorded during the Kraljevo M5.4 earthquake in Serbia was stochastic, in particular belonging to a class of linear stationary stochastic processes with Gaussian inputs or perhaps distorted by a monotonic, instantaneous, time-independent nonlinear function. However, because of the high frequency contents and rapid attenuation, near field blast motion spatially varies more significantly than earthquake ground motion [17], and so it is justified to examine this type of ground motion independently from earthquake induced vibrations. We note that the dynamics of ground motion induced by blasting has not yet been investigated by means of nonlinear time series analysis, even though this analysis was successfully applied in many other fields of research [18], including Earth and geophysical sciences [19]–[21]. These studies have proven that nonlinear time series analysis methods have vast potential in studying various types of experimentally recorded time series.

Besides the analysis of possible stochastic or deterministic nature of the recorded ground vibrations, we also develop a prediction model of PPV for the specific case study. Even though there are already many ground motion predictors, which could give a reasonable prediction of PPV, there is a justified need for updating the existing models by including PPV values of new recordings. Here, the prediction model is developed for the measurements of ground vibrations induced by blasting performed at limestone quarry “Suva Vrela” near Kosjerić, which is located in the western part of Serbia. The blasting was performed at five blasting locations, with a total of 426 blast holes, and with maximum 176–207 kg charge per delay. The explosive charge was detonated with a delay of 25 ms between each interval of blasting. The ground vibrations were measured at 13 monitoring points, placed at different distances from the blasting source. For the purpose of examining the possible presence of stochasticity in the recorded signal, we chose recordings at a single blasting location and three different measuring points. For every recording, we analyze all three components of the recorded velocity, namely the longitudinal, transversal and the vertical component. For the development of a reliable prediction model, however, we use a total number of 33 blast vibration records.

We chose limestone as a representative rock unit for investigating ground vibrations because it is the most common rock type in Serbian quarries, and also because limestone is the predominantly used rock type for civil engineering purposes. Moreover, blast-induced vibrations in limestone have been frequently investigated before, so that there is ample chance for comparing our findings with previous research. Kahriman [22] established an empirical relationship (with correlation coefficient *r* = 0.92) for the prediction of PPV at a limestone quarry in Istanbul, based on a scaled distance. Ozkahraman [23] applied a Kuznetsov equation to predict the mean fragment size from blasting limestone at Goltas quarry in Turkey. Kesimal et al. [24] investigated the impact of blast-induced ground motion on slope stability at Arakli-Tasonu limestone quarry in Trabzon (Turkey). Afeni and Osasan [25] studied the level of noise generated ground vibrations induced during blasting operations at the Ewekoro limestone quarry in Nigeria, and their effect on residential structures within villages near the quarry. Mohamed [26] developed an artificial neural network (ANN) model for PPV prediction in a limestone quarry in Egypt, by analyzing the predictive power of ANN with a different number of input units. Mohammadnejad et al. [27] used artificial neural networks and support vector machine for the prediction of blast-induced vibrations in two limestone quarries, obtaining rather high coefficient of determination (*R^2^* = 0.944).

Hence, the aim of our research is twofold: firstly, we want to confirm or reject the hypothesis that the strong ground motion is essentially stochastic and, secondly, we want to develop a site-specific prediction model of peak particle velocity (PPV). As we will show, by proving that the recorded signal is nondeterministic by nature, we turn to a single parameter of the recorded signal (the PPV) and use it to arrive at a reliable prediction model.

The setup of this paper is as follows. Section 2 provides a brief description of applied methodology and test procedures, including the blasting equipment, and the corresponding field work. In section 3, we perform surrogate data analysis, by testing the three null hypotheses on the stochastic nature of recorded blast-induced ground velocity. Next, we conduct a determinism test on the basis of the optimal embedding delay and the minimum embedding dimension, as determined for the examined time series. In section 5, we use existing conventional predictors, and evaluate their predictive power for the recorded PPV, after which we suggest a new model by using an artificial neural network approach. In the final section we summarize the main results and outline their possible implications, as well as give suggestions for further research.

## Methods

### 1. Field work

**Figure 1:**
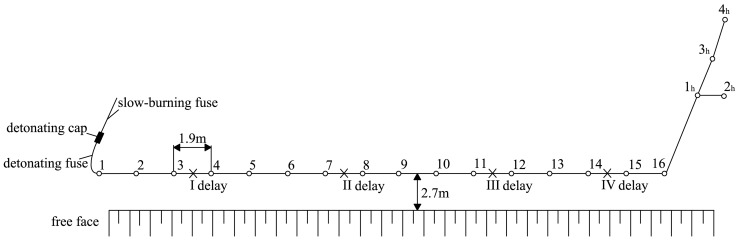
Distribution of blasting boreholes and the scheme of detonation order. There are four intervals of blasting, with delayed firing of 25

As noted above, for the purpose of conducting the surrogate data analysis, we examine recorded blast-induced ground motion at a single blasting location and 3 measuring points. The limestone quarry at “Suva Vrela” near Kosjerić, where the blasting was performed, is a permanently operating surface excavation site for extracting limestone, and it has been active for many years. Blasting is part of daily routine operations. No specific permissions were therefore required for the blasting studied in our paper. We also confirm that the field studies did not involve endangered or protected species. The blasting was performed in a single row, with 16 inclined and 4 horizontal boreholes. First 16 boreholes are inclined, in order to be parallel with the free face of quarry, while the last four boreholes are horizontal, in order to blast away the remnants of the rock that may be broken down by the preceding blasting. The distance between the inclined boreholes was 1.9–2.2 m, with depth between 17 and 19 m, while the length of horizontal boreholes was 1–3.5 m. The amount of explosives was in the range 30–62 kg for the inclined boreholes, and 1–7 kg for horizontal boreholes (Table 1). Four intervals of blasting were performed, with time delay between each interval of 25 ms, in order to avoid large rock disturbances caused by immediate explosion in all boreholes (Figure 1).

**Table 1:** Main technical characteristics of blasting boreholes at one mine location at “Suva Vrela” quarry. Data recorded at this site are used for surrogate data analysis.*

Borehole No.	Depth (m)	Amount of explosive (kg)	Borehole No.	Depth (m)	Amount of explosive (kg)
1	19	53	11	17	48
2	19	62	12	17	52
3	19	57	13	17	58
4	19	50	14	18	56
5	19	30	15	18	53
6	19	58	16	18	59
7	19	44	1_h_	1	2
8	19	58	2_h_	1	1
9	19	25	3_h_	3	7
10	17	35	4_h_	3	7

* Boreholes with index *h* are horizontal.

**Figure 2:**
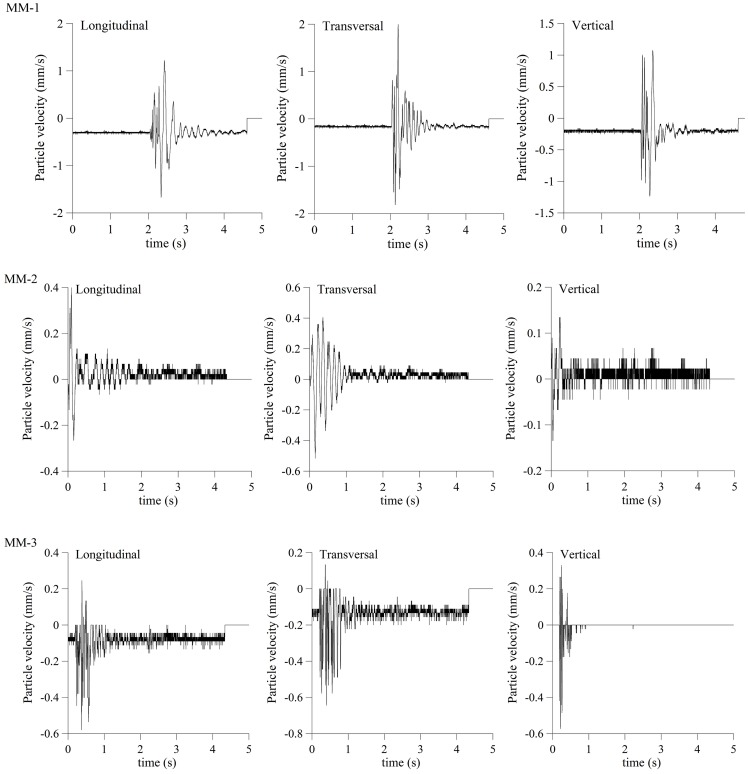
Longitudinal, transversal and vertical component of velocity time histories recorded at measuring points MM-1, MM-2 and MM-3.

The velocity of ground oscillation induced by blasting was measured by mobile seismograph of Vibralok type, with frequency range 2–250 Hz, sampling of 1000 Hz and trigger levels of 0.1–200 mm/s. The measuring was performed at three points, located at different distances from the blasting source (Table 2). Time series of blast induced ground velocities are given in Figure 2.

**Table 2:** Recorded ground velocity at three different distances from the blasting source for the borehole distribution given in Figure 1 and Table 1. *

Measuring point	Distance from the blasting source (m)	Total amount of explosive per interval (kg)	PPV_V_ (mm/s)	PPV_T_ (mm/s)	PPV_L_ (mm/s)
MM-1	546.191	182	1.123	1.990	1.670
MM-2	808.038	182	0.134	0.517	0.400
MM-3	1063.985	182	0.574	0.645	0.580

* Indices *V*, *T* and *L* stand for vertical, transversal and longitudinal component, respectively.

### 2. Analysis of stochasticity

Surrogate data analysis is performed by testing the three null hypotheses that the observed data belong to some class of linear systems: (a) data are independent random numbers drawn from some fixed but unknown distribution; (b) data originate from a stationary linear stochastic process with Gaussian inputs and (c) data originate from a stationary Gaussian linear process that has been distorted by a monotonic, instantaneous, time-independent nonlinear function [28]. In this paper, the surrogates are generated by using Matlab toolkit MATS [29], while the zeroth-order prediction error *ε* is calculated according to the algorithm in C suggested by Kantz and Schreiber [15].

The results of the surrogate data analysis are further confirmed by applying the determinism test [30], which is based on the assumption that if the time series originated from a deterministic system, the obtained vector field should consist solely of vectors that have unit length, indicating the average length of all directional vectors *κ* to be equal to 1. On the other hand, for a completely random system, *κ*≈0 [31].

For calculating the optimal embedding delay, we use average mutual information method [32], which utilizes the first local minimum of mutual information as optimal embedding delay. In order to determine the minimal required embedding dimension *m*, we use the procedure suggested in [33] that identifies the number of “false nearest neighbors”, points that appear to be the nearest neighbors because the embedding space is too small. We use the criterion which utilizes the fact that the normalized distance between the embedding coordinates of two presumably neighboring points is larger than a given threshold (*R_tr_*), if these two points are false neighbors. According to Kennel et al. [33], the value of *R_tr_* = 10 proves to be a good choice for most data sets.

### 3. Prediction models

Prediction models are evaluated using the existing conventional predictors [2], [6], [11]–[14] and ANN approach. Various conventional predictors proposed by different researchers are given in Table 3 [2], [6], [11]–[14]. In present paper, we use feed-forward multi-layer perceptron, frequently applied for modeling the blast-induced vibrations [34], [35]. This type of neural network usually consists of three layers: input, hidden and output layer. Among various algorithms available for training ANN, we used the back-propagation training rule optimized by Broyden–Fletcher–Goldfarb–Shannon (BFGS) algorithm, which is considered to be one of the best of quasi-Newtons technique, that is error tolerant, yields good solutions and converges in a small number of iterations [36]. The computational advantage of BFGS over back-propagation especially holds for small to moderate sized problems [37], which is the case in present analysis [34], [35], [38].

**Table 3:** Different conventional predictors.*

Conventional predictor	Equation
Duvall-Petkof (USBM) (1959)	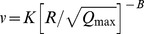
Langefors-Kihlstrom (1963)	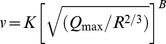
General predictor (1964)	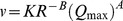
Ambraseys-Hendron (1968)	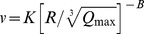
CMRI (1993)	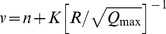

* *v* is the peak particle velocity (PPV) in mm/s, *Q_max_* is the maximum charge per delay, in kg, *R* is the distance between the blasting source and measuring point, in meters, and *K*, *B*, *A*, and *n* are site constants.

Performances of different predictor models were estimated using standard statistical evaluation criteria given in Table 4 [39].

**Table 4:** Statistical error parameters used for models' evaluation. *

Statistical parameter	Equation
Mean absolute percentage error	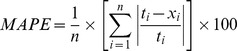
Root mean square error	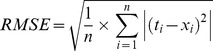
Variance absolute relative error	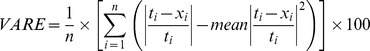
Median absolute error	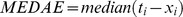
Variance account for	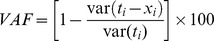

* *t_i_* represents measured value of PPV, while *x_i_* denotes predicted value of PPV.

## Results and Discussion

### 1. Surrogate data analysis

The testing of the first null hypothesis is performed in the following way. We generate 20 surrogates by randomly shuffling the data (without repetition), thus yielding surrogates with exactly the same distribution yet independent construction. Then, in order to compare the original data and generated surrogates, we calculate the zeroth-order prediction error *ε* [28]. If the zeroth- order prediction error for the original recordings (*ε_0_*) is smaller in comparison to the calculated error for surrogate data (*ε*), then a null hypothesis can be rejected. On the other hand, if *ε_0_*>*ε* at any instance of the test, the null hypothesis is confirmed. Usually, more than one wrong result out of 20 is not considered acceptable [40]. In all cases, *ε_0_* is smaller than *ε* which allows us to reject the null hypothesis, with significance level of 95%.

**Figure 3:**
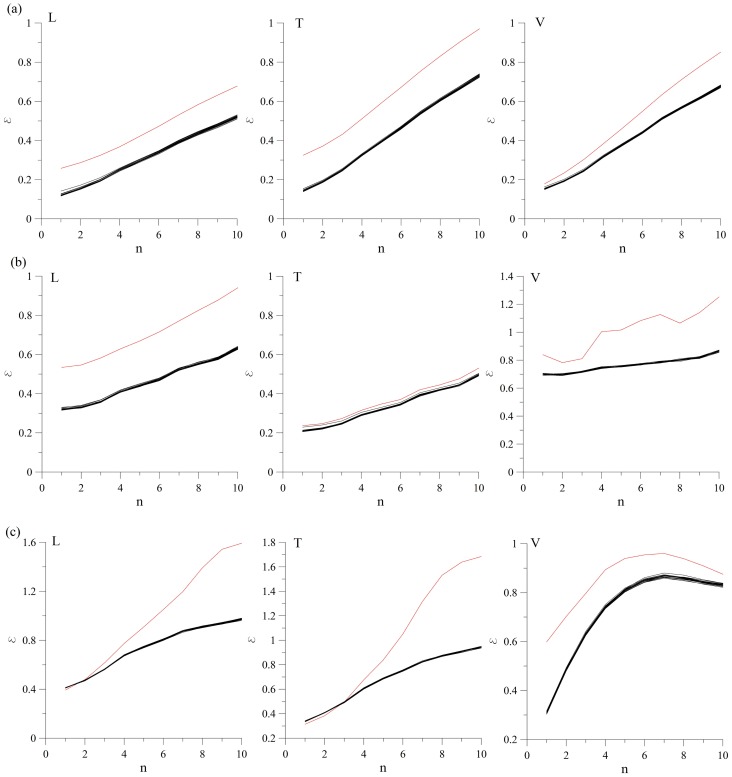
Surrogate data test for the second null hypothesis. Zeroth-order prediction error for the ground velocity recordings at the following measuring points: (a) MM-1 (L, T and V), (b) MM-2 (L, T and V), (c) MM-3 (L, T and V). In all the examined cases, *ε_0_*>*ε*, so the null hypothesis cannot be rejected in neither of the examined velocity recordings. Red line denotes the zeroth-order prediction for the original time series (*ε_0_*), and black lines denote zeroth-order prediction for the surrogates (*ε*). Abbreviations L, T and V stand for longitudinal, transversal and vertical component of the recorded ground velocity, respectively.

For purpose of testing the second null hypothesis, we employ the phase randomization analysis [41]. The results are shown in Figure 3. Obviously, we could not reject the null hypothesis (at 95% significance level) since *ε_0_*>*ε* for all the tested surrogates.

**Figure 4:**
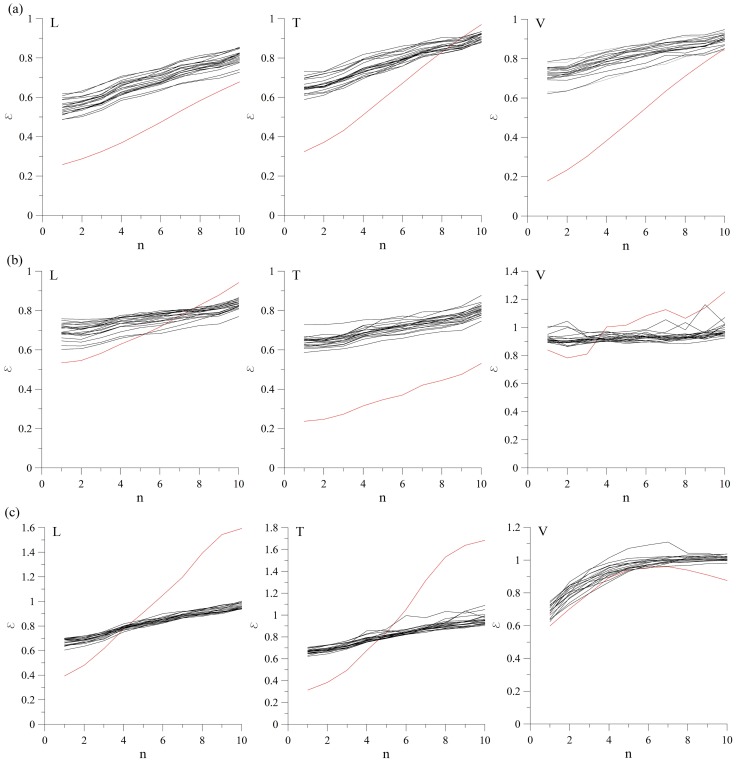
Surrogate data test for the third null hypothesis (AAFT). Zeroth-order prediction error for the ground velocity recordings at the following measuring points: (a) MM-1 (L, T and V), (b) MM-2 (L, T and V), (c) MM-3 (L, T and V). It is clear that *ε_0_*>*ε* for the vertical velocity component at MM-2, and for the longitudinal and transversal velocity component at MM-3, for prediction steps *n*>4. In all the other cases, *ε_0_<ε*, allowing us to reject the null hypothesis. Red line denotes the zeroth-order prediction for the original time series, and black lines denote zeroth-order prediction for the surrogates.

In order to test the third null hypothesis, we calculate the amplitude adjusted Fourier-transformed (AAFT) surrogates [41]. As in the previous case, we generated 20 surrogates for each of the observed cases and calculated prediction error *ε*. Interesting results appear for the vertical velocity component at MM-2, and for the longitudinal and transversal velocity component at MM-3, where *ε_0_*>*ε* for prediction steps *n*>4 (Figure 4). This kind of prediction behavior could result from the very nature of the applied method itself, since the generation of amplitude adjusted surrogates results in changes to the power spectrum of the final surrogate, which further causes the power spectrum whitening of the original data [42].

**Figure 5:**
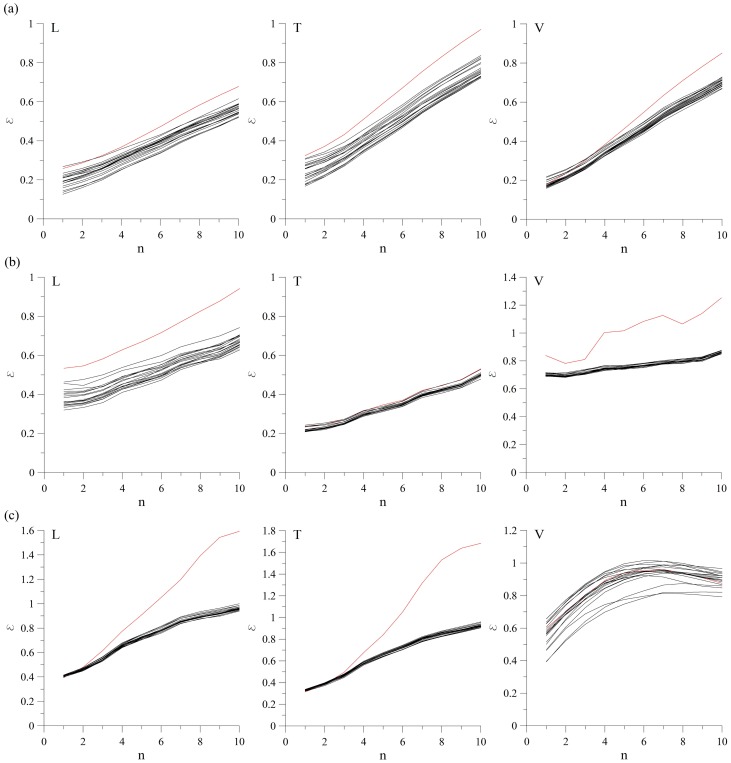
Surrogate data test for the third null hypothesis (iterated AAFT surrogates). Zeroth-order prediction error for the ground velocity recordings at the following measuring points: (a) MM-1 (L, T and V), (b) MM-2 (L, T and V), (c) MM-3 (L, T and V). In all the examined cases, except for the vertical velocity component at MM-3, *ε_0_*>*ε*, so the null hypothesis could not be rejected for all of the examined velocity recordings. Red line denotes the zeroth-order prediction for the original time series, and black lines denote zeroth-order prediction for the surrogates.

In order to exclude the possible influence of the method itself on the final result, AAFT method could be further improved by performing a series of iterations in which the power spectrum of AAFT surrogate is adjusted back to that of the original data before the distribution is rescaled back to the original distribution (iterated AAFT method). This is obtained by adjusting back the amplitudes of the Fourier transformed AAFT surrogates to the Fourier transformed surrogates of the rescaled original data. The obtained surrogates are then inverse Fourier transformed and rescaled back to the original data distribution by sorting the original data according to the ranking of the Fourier-transformed surrogate [42]. These two steps are iterated for several times (in our case 500), until the whitening of the power spectrum becomes sufficiently small. As in the previous cases, we generated 20 such surrogates and calculated zeroth-order prediction error *ε* (Figure 5). It is clear that in case of vertical velocity component measured at MM-3, *ε_0_* is well within ε for several surrogates, so the null hypothesis could be rejected. In all other cases, *ε_0_*>*ε*, so we could not reject the null hypothesis.

### 2. Determinism test

In order to apply this test, the observed scalar series are embedded into the appropriate phase space according to Takens [43]. The values of optimal embedding delays are:

*τ* = 46, *τ* = 35 and *τ* = 49 for longitudinal, transversal and vertical component of velocity recordings at MM-1, respectively,
*τ* = 43, *τ* = 44 and *τ* = 38 for longitudinal, transversal and vertical component of velocity recordings at MM-2, respectively,
*τ* = 56, *τ* = 59 and *τ* = 129 for longitudinal, transversal and vertical component of velocity recordings at MM-3, respectively.

On the other hand, the results of false nearest neighbor technique showed that fraction of false nearest neighbors rises with the increase of embedding dimension, which could indicate high level of stochasticity in the input data.

**Figure 6:**
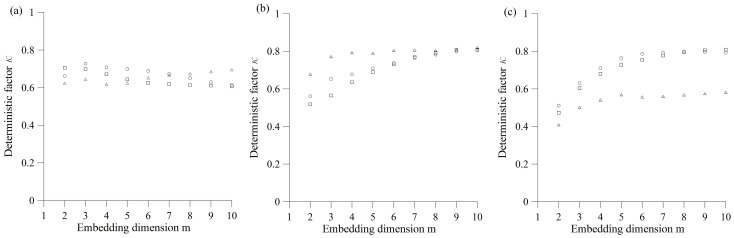
Determinism test for velocity recordings at measuring points: a) MM-1, b) MM-2 and c) MM-3. Squares, circles and triangles denote longitudinal, transversal and vertical component of the velocity, respectively. The values of determinism factor *κ* are given for the embedding dimension in range *m* = 2–10. It is evident that *κ*≤0,81, indicating the absence of deterministic behavior.

For the purpose of employing the determinism test, we examined velocity recordings for different values of embedding dimension, since embedding dimension is needed as input parameter for deterministic test (Figure 6). In order to calculate the determinism factor *κ*, we included only those boxes visited at least one time by the trajectory. As apparent from Figure 6, the value of determinism factor *κ* is in the range 0.4–0.81, indicating the absence of determinism in observed ground motion.

### 3. Prediction models

#### 3.1. Prediction of PPV using conventional predictors

Since we established the fact that the recorded ground motion is nondeterministic by nature, and, consequently, impossible to predict, we turn to common empirical attenuation equations, which represent prediction models for PPV as a function of scaled distance [5]. These equations are developed on the basis of the assumption that the total energy of the ground motion generated by blasting varies directly with the weight of detonated explosives and that it is inversely proportional to the square distance from blasting point. These empirical models often proved as a reliable choice for PPV prediction, even though ground motion data scatter significantly. Also, some of the existing vibration standards for preventing the structural safety use scaled distance as a damage criterion [44].

**Figure 7:**
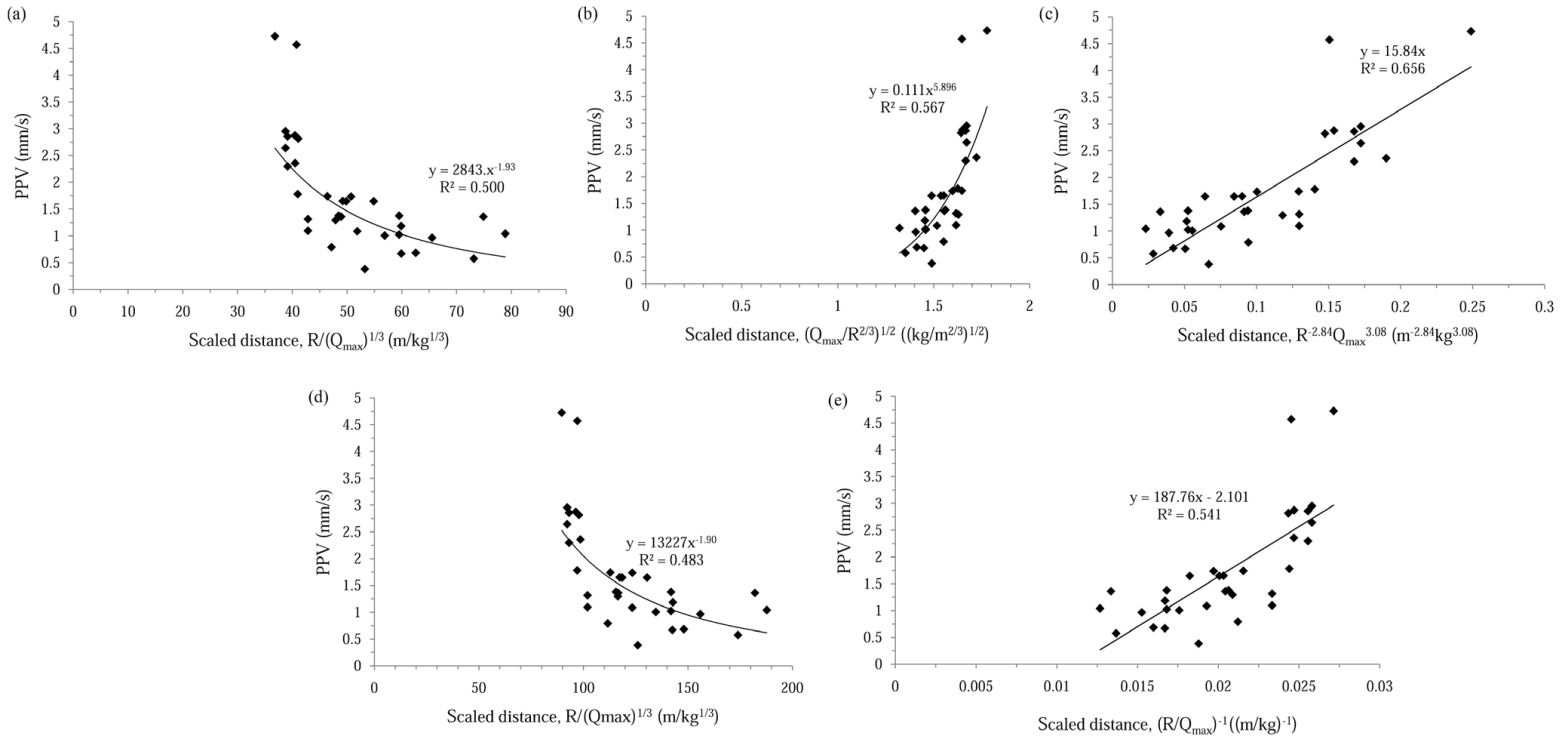
Resultant PPV versus scaled-distance relationship for different conventional predictors: (a) USBM, (b) Langefors-Kihlstrom, (c) General predictor, (d) Ambraseys-Hendron, (e) CMRI. Note that coefficients A and B for General predictor were determined using multiple regression approach.

**Figure 8:**
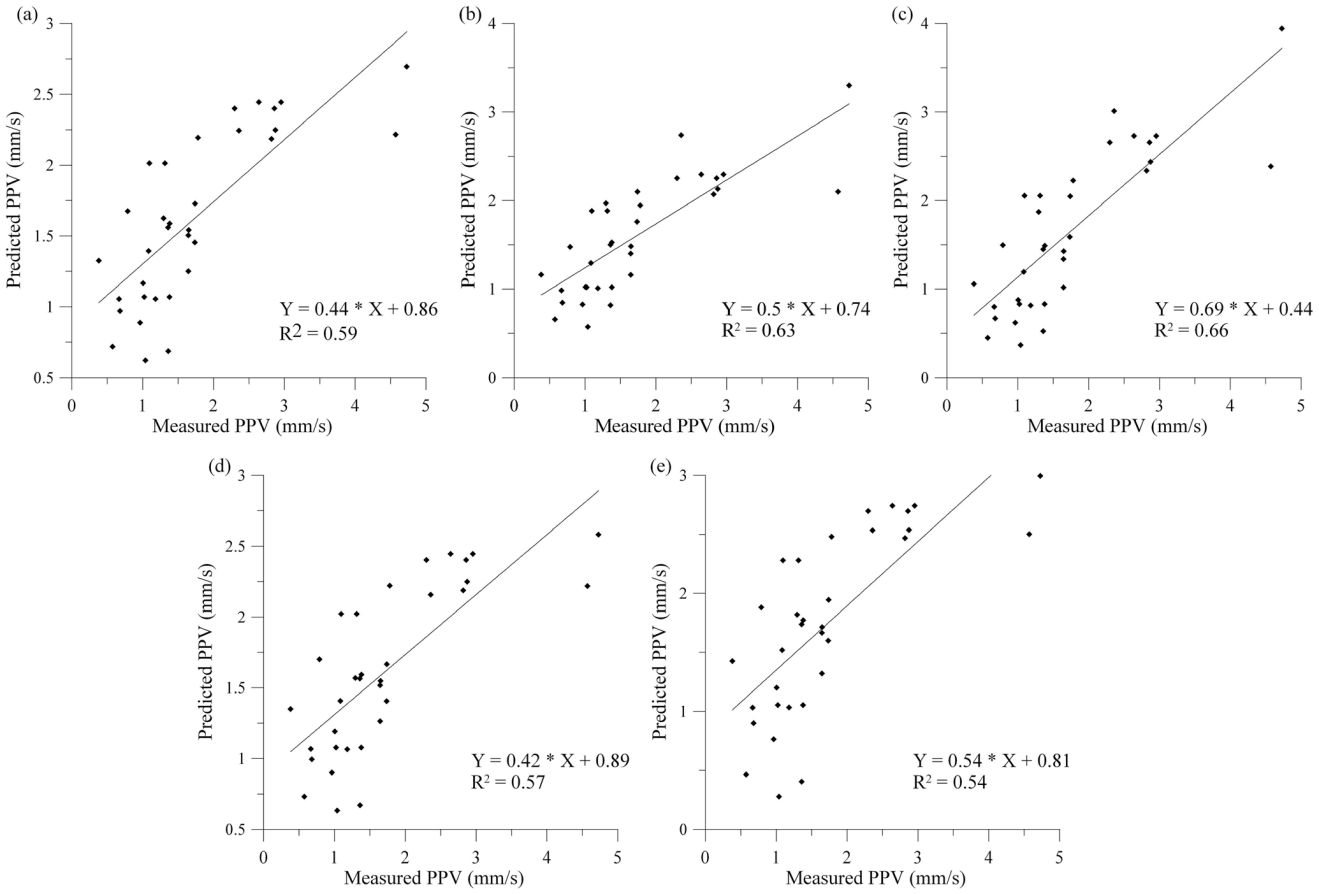
Measured PPV vs. predicted PPV by conventional predictors: (a) USBM, (b) Langefors-Kihlstrom, (c) General predictor, (d) Ambraseys-Hendron, (e) CMRI. It is clear that each of the predictor gives rather low coefficient of determination, in the range *R^2^* = 0.54–0.66.

In present analysis, the site constants were determined from the multiple regression analysis of the previously mentioned 33 recordings (Figure 7 and Table 5). The relationship between measured and predicted PPV by conventional predictor equations is given in Figure 8. As it can be seen, in case of using conventional predictors for estimating PPV, coefficient of determination (*R^2^*) is varying between 0.54 (CMRI) and 0.66 (General predictor).

**Table 5:** Calculated values of site constants for conventional predictors.

Equation	K	B	A	n
Duvall-Petkof (USBM) (1959)	2843	1.93	/	/
Langefors-Kihlstrom (1963)	0.11	5.90	/	/
General predictor (1964)	15.84	2.84	3.08	/
Ambraseys-Hendron (1968)	13227	1.90	/	/
CMRI (1993)	187.76	/	/	−2.10

#### 3.2. Prediction of PPV using the artificial neural network approach

Preceding analysis showed that conventional methods cannot give accurate prediction of PPV, which could be explained by the fact that these models are approximate, treating blast induced ground vibrations in dependence only on maximum charge per delay or distance to the blasting source, neglecting a number of other influential parameters, like total charge, stemming, hole depth, physical-mechanical properties of rock mass or explosive characteristics [34]. Since the number of affecting parameters is large and the relations among them could be very complex and often unknown, empirical methods may not be always suitable for accurate prediction of PPV. Also, the existing empirical attenuation relations often result from one-location database, and cannot predict PPV value with satisfying accuracy at other blasting locations, mainly due to heterogeneous and anisotropic rock mass properties. In order to overcome these obstacles of conventional predictors, artificial neural network approach (ANN) is frequently implemented, mainly because of its ability to deal with large number of different patterns, to learn by examples and to detect similarities in inputs, even though they may have never been known previously.

ANN has been successively used in the area of blast-induced vibrations so far. Khandelwal and Singh [45] predicted the PPV by ANN, taking into consideration the distance from the blast face to measuring point and explosive charge per delay. They compared their findings with the commonly used vibration predictors and their results were more accurate by ANN prediction as compared to vibration predictor equations. Khandelwal and Singh [34] developed a three-layer feed-forward back-propagation neural network for predicting the PPV and frequency and obtained a much higher coefficient of determination (*R^2^* = 0.98) in comparison to the conventional predictors (*R^2^* = 0.13–0.54). Monjezi et al. [35] also developed a feed-forward back-propagation neural network model, with 4 input parameters, two hidden layers and one output parameter (PPV). The accuracy of prediction by using ANN was much higher (*R^2^* = 0.95) in comparison to the conventional predictors or mutlivariate regression analysis (*R^2^* = 0.38–0.80). In present paper, we use identical approach as in [40], including average hole depth as an additional input unit (Table 6).

**Table 6:** Input-output parameters for the ANN training and their range.

Type of data	Parameter	Range
Input unit 1	Total charge, Q_t_ (kg)	815–4675
Input unit 2	Maximum charge per delay, Q_i_ (kg)	176–247
Input unit 3	Distance from blasting source (m)	521.552–1077.28
Input unit 4	Average hole depth (m)	8.25–18
Output unit	Peak particle velocity (mm/s)	0.381–4.728

Before we proceed to further analysis, one should note that development of ANN model for small data sets is not an exception. Mohamadnejad, et al. [27] also examined small data set (37) using support vector machine algorithm and regression neural network, obtaining rather high prediction accuracy (*R^2^* = 0.92). Moreover, Monjezi et al. [39] developed a four-layer feed-forward back-propagation neural network, using only 20 data sets. In this case, high prediction accuracy was also obtained (*R^2^* = 0.927).

In order to develop a valid ANN model for PPV prediction, input data have to be preprocessed, due to their different nature and unit. Regarding this, all the input and output parameters were scaled between 0 and 1. This was done to utilize the most sensitive part of neuron and since output neuron being sigmoid can only give output between 0 and 1 [35]. The scaling of output parameter was done in the following way:
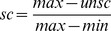
where *sc* and *unsc* stand for the scaled and unscaled values, and *max* and *min* represent the maximum and minimum value of the parameter, respectively.

After analyzing several cases of networks with various numbers of hidden layers and hidden neurons, the most precise model for PPV prediction was obtained by neural network with one hidden layer and one hidden unit. The learning rate (0.1) and momentum coefficient (0.9) were chosen by trial and error, leading to the minimum prediction error [26], [46]. The total data set comprising 33 points has been divided as follows: 65% of the data for training, and 35% for testing and validation, that were not used for training. In other words, training of the network was carried out using 22 cases and testing and validation of the network was performed using 11 different cases.

**Figure 9:**
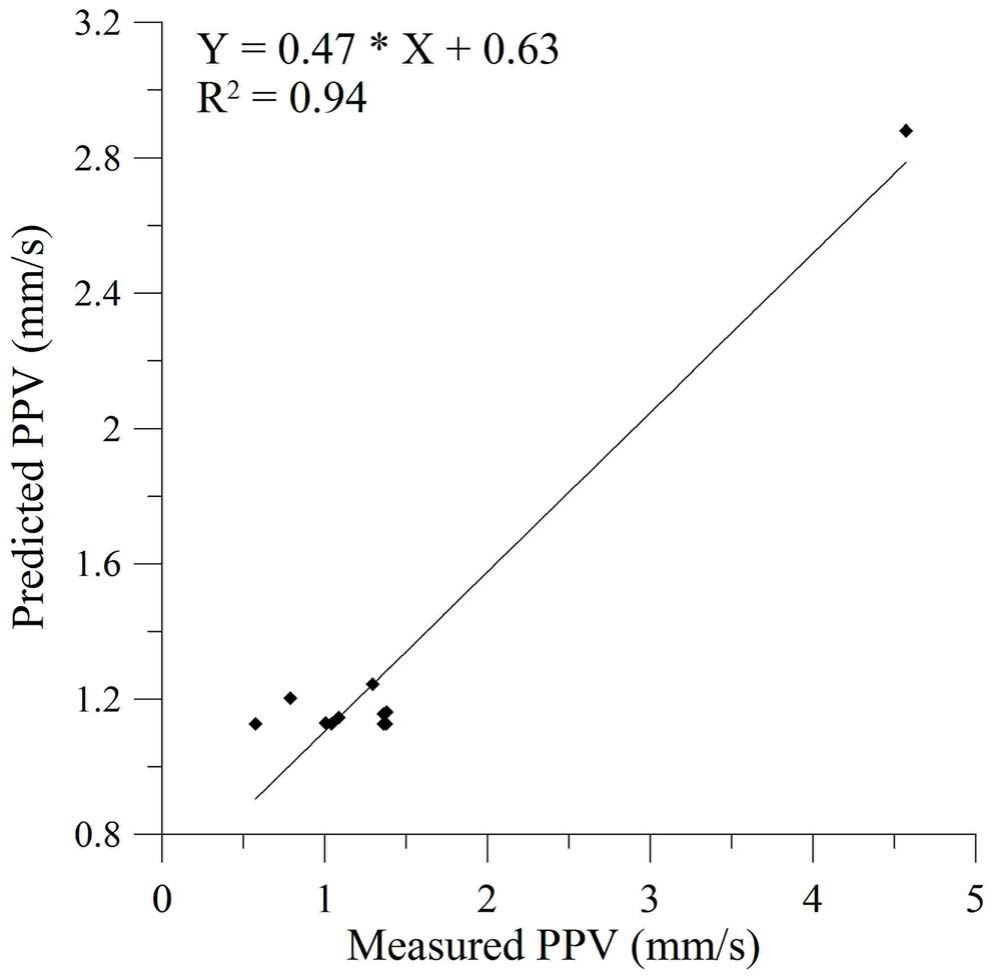
Measured PPV vs. predicted PPV by ANN predictor, with high coefficient of determination (*R^2^* = 0.94).

In order to evaluate the model performance, we determined the correlation between the predicted and real measured values of PPV. High value of coefficient of determination (*R^2^* = 0.94) demonstrates good performance of the proposed network (Figure 9). The lowest Mean Squared Error (MSE = 0.4489) was obtained after 2500 epochs of training.

**Figure 10:**
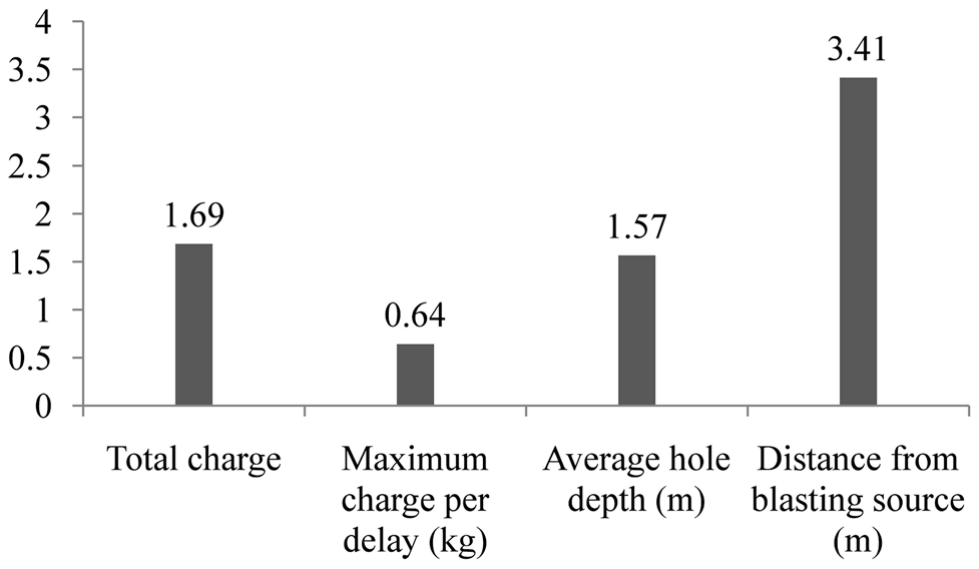
Global sensitivity analysis of input parameters.

The performed analysis could be further expanded, by inspecting the impact of each input parameter separately on the final value of PPV. This could be achieved by applying sensitivity analysis, which represents a method that enables us to determine the effectiveness of each input unit on the final value of output parameter [39]. Global sensitivity analysis, which was carried out for all the input parameters, indicated that the distance from blasting source has the strongest impact on the PPV value (Figure 10), corresponding well with the previous research on this topic [34]–[35], [39]. We note that the impact of other factors (total charge, maximum charge per delay and average hole depth) was not evaluated in detail.

#### 3.3. Evaluation of the models' performance

**Figure 11:**
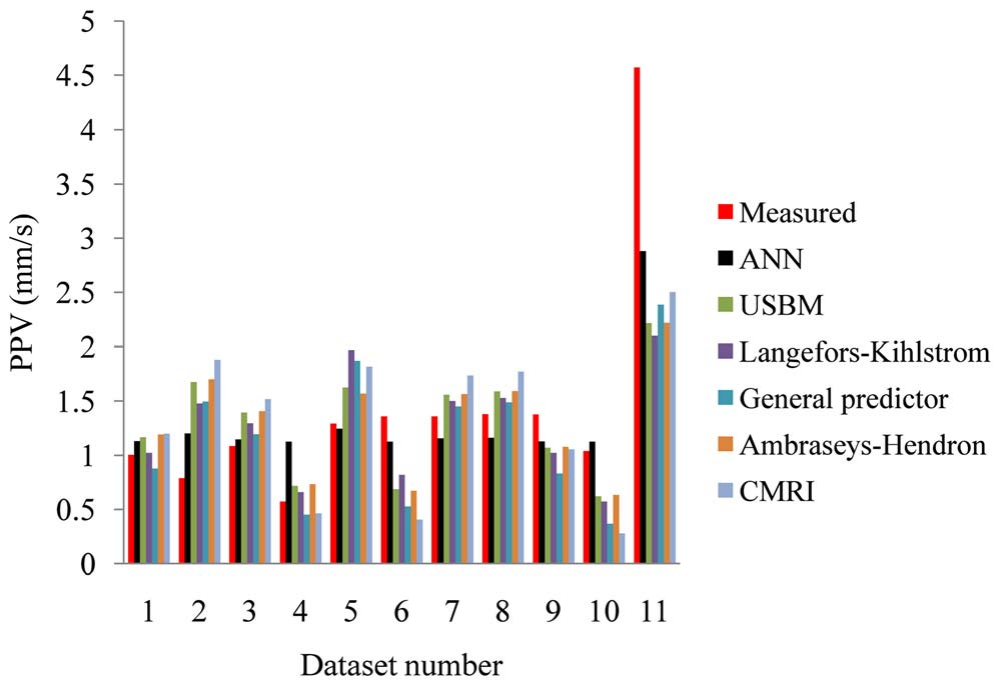
Comparison of predicted PPV by different predictors. Abbreviations AH, GP and LK stand for Ambraseys-Hendron, General Predictor and Langefors-Kihlstrom, respectively.

If we compare the values of PPV predicted by different methods (conventional predictors and ANN), it is clear that prediction by ANN is closer to the measured PPV, whereas predictions by conventional predictors have wide variation (Figure 11).

Calculated statistical errors are given in Table 7. It is clear that ANN has the lowest values of MAPE, VARE, MEDAE and RMSE, while it has the highest value of VAF, in comparison to conventional predictors.

**Table 7:** Performances of different models for predicting PPV using statistical error parameters given in Table 4.

Model	MAPE	VARE	MEDAE	VAF	RMSE
Duvall-Petkof (USBM) (1959)	50.2	47.62	0.31	65.5	0.96
Langefors-Kihlstrom (1963)	45.19	43.09	0.36	60.56	0.99
General predictor (1964)	50.87	48.29	0.55	0.38	0.94
Ambraseys-Hendron (1968)	50.83	48.15	0.30	65.41	0.96
Singh-Roy (CMRI) (1993)	65.75	61.47	0.43	73.87	0.99
ANN	35.29	33.61	0.22	81.60	0.67

## Conclusions

There is a justified need for updating the attenuation equations by including PPV values of new shot records into analysis data, due to complexity of the geological and technological parameters that affect blasting. Apparently, the available empirical attenuation equations, proposed on the basis of measured ground motion at one site, are not accurate enough to predict the ground vibration level at other locations. Therefore, it is important to include new data in the analysis, in order to develop an appropriate attenuation model. In this case, the performed analysis of the blast induced ground vibrations at limestone quarry “Suva Vrela” resulted in the following:

we showed that the ground vibrations due to blasting are stochastic in nature. More precisely, they belong to class of stationary linear stochastic processes with Gaussian inputs, which could be distorted by a monotonic, instantaneous, time-independent nonlinear function. The basic method used in this paper, in search for possible stochastic behavior, was surrogate data analysis [15], [40]–[42]. Testing of the three null hypotheses indicates the stochasticity as an important factor in blast induced ground motion. The results of surrogate data analysis were further confirmed by the application of a determinism test [30]. Rather low values of the determinism factor (*κ*<0.81) indicate the possible absence of determinism in the recorded ground motion. In this way we proved that the recorded signal does not belong to deterministic systems and, hence, cannot be simulated or predicted by using theoretical or empirical formulas;
we developed a site-specific prediction model of PPV. After we had shown that conventional predictors cannot give a satisfactory level of prediction accuracy (*R^2^* = 0.54–0.66), we chose to train a neural network, and obtained very high level of prediction accuracy (*R^2^* = 0.94), with satisfying level of statistical errors, in comparison to the conventional predictors. Furthermore, global sensitivity analysis showed that distance from blasting source (*R*) has the strongest impact on the final value of PPV in comparison to the other three input parameters.

Hence, there are two main conclusions of our analysis. Firstly, our investigation strongly suggests that blast induced ground motion represents a linear stochastic process, which corresponds well with the results of our previous work on earthquake ground motion [16]. Secondly, we developed a prediction model with high accuracy, which is the first known ANN model for the blast-induced ground vibration recorded in Serbia.

However, we have to emphasize that the results of this analysis are valid only for the blast induced ground velocity recorded at the limestone quarry “Suva Vrela” near Kosjerić in western part of Serbia. The question of possible determinism in blast induced ground vibrations still remains open for different rock masses (igneous, metamorphic or some other sedimentary) and for different blast parameters (maximum charge per delay, hole depth, explosive characteristics, etc). Moreover, various geotechnical parameters (compressive and tensile strength, Young's modulus, Poisson ratio, etc) could considerably affect the blast induced ground motion even within the same rock unit. It would be interesting to investigate possible stochastic nature of ground motion in different surroundings and by varying blast parameters. Only in that way, by comparing these events, and, in the same time, by opposing the results of the research in different areas, could the general nature of the blast induced ground motion be determined.
